# Theranostic Innovative Strategies for Brain Diseases: New Insights on Neurovascular Unit-Associated Pathological Changes in Neurodegenerative Disorders and Aging

**DOI:** 10.3390/ijms27167165

**Published:** 2026-08-11

**Authors:** Giulia Terribile, Matilda Pedrinazzi, Irene Frigerio, Giulio Sancini, Romina Combi

**Affiliations:** 1 School of Medicine and Surgery, University of Milano-Bicocca, Via Cadore 48, 20900 Monza, Italy; giulia.terribile@unimib.it (G.T.); m.pedrinazzi@campus.unimib.it (M.P.); i.frigerio11@campus.unimib.it (I.F.); giulio.sancini@unimib.it (G.S.); 2 Milan Centre for Neuroscience (NeuroMI), University of Milano-Bicocca, 20126 Milan, Italy; 3 PhD Program in Neuroscience, University of Milano-Bicocca, Via Cadore 48, 20900 Monza, Italy; 4 NanoMedicine Center (NANOMIB), University of Milano-Bicocca, 20126 Milan, Italy

**Keywords:** neurovascular unit, neurodegenerative disorders, aging, theranostic, nanomedicine

## Abstract

Central nervous system (CNS) disorders represent a significant healthcare challenge, with aging as the primary risk factor. Current clinical management remains predominantly symptomatic, as late-stage diagnosis and the blood–brain barrier (BBB) limit therapeutic efficacy. This review synthesizes emerging innovations in neurotheranostics—integrated diagnostic and therapeutic platforms—focusing on the neurovascular unit (NVU) as a central pathogenic driver and target. Evidence indicates that NVU and BBB dysfunction are early events in Alzheimer’s, Parkinson’s, amyotrophic lateral sclerosis, and Huntington’s diseases, often preceding classic neuropathological hallmarks. The review highlights the potential of nanotechnology, engineered nanoparticles (NPs) and microRNAs (miRNAs) as precision tools for early detection and targeted CNS delivery. Additionally, it discusses the transformative impact of artificial intelligence (AI) in facilitating personalized, predictive care. Transitioning from a generic “one-pill-for-one-disease” model to a patient-centered strategy targeting early NVU alterations is essential. Integrating AI, nanotechnology and NVU-focused strategies offers a promising path toward effective, personalized disease-modifying therapies.

## 1. State of the Art of Current Approaches and Urgent Needs

The concept of theranostics—the integration of diagnostic and therapeutic functionalities within a single platform—has emerged as a transformative paradigm in modern medicine [1]. Originally introduced by Funkhouser in 1998 and initially developed in the context of oncology, theranostics has progressively expanded its scope to encompass one of the most challenging frontiers in medicine: central nervous system (CNS) disorders, particularly in the context of neurodegenerative diseases (NDs) [2].

NDs, including Alzheimer’s disease (AD), Parkinson’s disease (PD), amyotrophic lateral sclerosis (ALS) and Huntington’s disease (HD), collectively represent one of the greatest healthcare challenges of the twenty-first century, imposing a substantial social and economic burden through long-term disability, loss of independence and increasing demands on healthcare and caregiving systems [3]. As global economic development and healthcare infrastructures improve, there is a corresponding rise in the incidence of neurodegenerative disorders. This trend is demonstrated by Dorsey et al. [4]. Although age-standardized mortality and disability-adjusted life year (DALY) rates associated with neurological disorders have declined over recent decades, the absolute burden continues to rise and is projected to increase further as populations age worldwide [3]. Also, stroke and migraine rank among the top global causes of disease burden measured by DALYs, because they stem from neurovascular unit (NVU) dysfunction [5]. These two conditions show how blood–brain barrier (BBB) damage hurts world health right now, while long-term failure causes slow brain diseases over time. Indeed, the NVU keeps the brain stable by supporting the BBB and controlling blood flow. Damage to the NVU causes brain inflammation, nerve cell damage and barrier leaks in many brain diseases. This trend largely reflects the strong age dependence of NDs, whose incidence and prevalence increase markedly in later life as a consequence of cumulative molecular and cellular damage, impaired repair mechanisms, chronic inflammation and progressive loss of physiological resilience [6].

However, neurodegeneration should not be viewed solely as a disease of aging. Rather, the pathological processes that eventually manifest in late life may result from a lifelong accumulation of risk factors, with particular relevance for exposures occurring during critical windows of susceptibility. Among these, the neurodevelopmental period represents a highly vulnerable stage during which environmental perturbations can induce persistent alterations in brain structure and function, potentially influencing disease susceptibility decades later [7,8]. Increasing evidence supports a life-course model of neurodegeneration in which late-life neurological decline emerges from the cumulative interaction between genetic predisposition and environmental exposures acting throughout life.

Considering genetics, NDs are influenced by a wide range of factors, from straightforward direct predisposition in diseases like Huntington’s disease to more complex roles in diseases like AD and PD. Several causative genes inherited as Mendelian traits have been identified for familial forms, which are characterized by the presence of rare variants with strong effect sizes. However, only a small percentage of ALS, AD, and PD cases (5–10%) are familial, while the majority are non-familial cases with late onset forms for which GWAS studies identified the existence of thousands of common variants that are associated with an increased risk of disease and highlighted the heterogeneity of these disorders. Even among cases attributed to the same high-penetrance mutation, considerable heterogeneity in penetrance, age at onset, and disease trajectory is reported, implying that other genetic modifiers or environmental elements interact with the primary pathogenic variant to affect phenotypic manifestation. A paradox of differing pathological outcomes for the same genetic mutation seems to emerge. This paradox suggests that the mutations do not actively “cause” the pathology but lead to an inadequate response to damage: the mutant allele exposes a failure in damage response, but the precise pathology will depend on the type of damage. This hypothesis correlates well with the discovery that the majority of identified disease loci are generally components of damage response and clearance mechanisms, with the deposited proteins revealing the precise failing mechanism. This observation explains why the phenotype of the pathology is age-dependent and related also to environment-induced damage deriving from other overloaded pathways. In this complex model of pathogenesis, environmental factors play a role in disease initiation, and the reported incomplete penetrance is the reflection of both genetic and environmental components.

The environmental exposures encompass a broad range of physical, chemical, metabolic and inflammatory stressors that may exert their greatest impact during critical windows of heightened susceptibility, particularly neurodevelopment and aging. Despite their heterogeneity, many of these factors appear to converge on common pathogenic pathways involving dysfunction of brain barrier systems, including the BBB and the NVU [9,10]. Within this framework, alterations of BBB integrity represent a plausible mechanistic link between early-life and age-related determinants of neurodegeneration. Disruption of neurovascular and neuroimmune homeostasis during neurodevelopment may establish latent vulnerabilities that remain clinically silent for decades, whereas progressive barrier deterioration during aging can facilitate the entry of peripheral inflammatory mediators, circulating toxicants and other blood-borne factors into the CNS. Together, these processes may progressively erode CNS resilience, lowering the threshold for neurodegenerative disease initiation and progression [11].

Together, these observations support the view that BBB dysfunction may contribute both to the early establishment of vulnerability and to the later-life manifestation and progression of neurodegenerative disease.

Despite growing knowledge related to pathophysiological processes and decades of intensive research, disease-modifying therapies remain largely elusive, and current clinical management is predominantly symptomatic. A critical bottleneck in therapeutic development lies in the late-stage diagnosis of these conditions: by the time clinical symptoms manifest, irreversible neuronal loss has already occurred, rendering intervention substantially less effective [12,13].

Moreover, beyond the challenge of late diagnosis and intervention, the BBB represents a major obstacle to the development of effective disease-modifying therapies for neurodegenerative disorders. While its primary physiological function is to protect the brain parenchyma from blood-borne agents, the BBB simultaneously restricts the penetration of most therapeutic compounds into the CNS, limiting their ability to reach target sites at therapeutically relevant concentrations. This highly selective gatekeeping function therefore remains another critical bottleneck in CNS drug development [14]. Importantly, the challenge extends beyond drug delivery itself. Aging and NDs are frequently associated with structural and functional alterations of BBB integrity, resulting in region-specific and disease-dependent changes in permeability. Such alterations can profoundly affect drug biodistribution, target engagement and clearance, thereby complicating dose optimization and therapeutic efficacy. At the same time, an impaired barrier may increase exposure of vulnerable neural tissues to circulating drugs and their metabolites, potentially exacerbating off-target effects and toxicity [15]. Consequently, a deeper understanding of BBB dysfunction across the lifespan and during disease progression is essential not only for improving CNS drug delivery but also for predicting therapeutic responses and minimizing adverse effects.

In this context, recent advances in nanotechnology, molecular imaging and precision medicine have accelerated the development of theranostic platforms capable of simultaneously identifying pathological processes and delivering targeted therapies. In the neurological field, these innovations have given rise to neurotheranostics, an emerging discipline that integrates diagnosis, monitoring and treatment within a unified framework [16].

Among the most promising approaches are engineered nanoparticles (NPs) designed to overcome the highly selective nature of the BBB. By exploiting tailored physicochemical properties and targeting ligands, NPs can facilitate the transport of therapeutic and imaging agents across the BBB, selectively target specific cell populations, and enable real-time monitoring of disease progression and treatment response. As such, nanoparticle-based platforms offer a unique opportunity to address both the diagnostic and therapeutic limitations that currently hinder effective management of neurodegenerative disorders [17].

By integrating diagnostic and therapeutic functionalities into a single platform, neurotheranostics has the potential to redefine the clinical management of neurological diseases. This paradigm could enable earlier disease detection, more precise patient stratification, personalized treatment selection and continuous monitoring of therapeutic efficacy [16]. Ultimately, such an approach may streamline patient management, reduce the need for repeated hospital visits and complex treatment protocols, improve patient compliance, and enhance the accessibility and cost-effectiveness of neurological and neurosurgical care.

Despite these promising advances, substantial barriers remain before neurotheranostic technologies can be translated into clinically viable and economically sustainable solutions. Challenges related to biological safety, long-term biodistribution, BBB penetration efficiency, large-scale manufacturing, regulatory approval and clinical validation continue to limit widespread implementation [18]. Nevertheless, neurotheranostics represents a transformative strategy with the potential to bridge the longstanding gap between diagnosis and treatment, paving the way toward truly personalized neurological care.

The present review aims to synthesize current knowledge and emerging innovations at the intersection of neurotheranostics and NVU physiology in the context of NDs and aging. We discuss how advances in nanotechnology, biomarker discovery, molecular imaging and targeted drug delivery are converging to create integrated strategies capable of simultaneously detecting and modulating NVU-associated pathological changes. Particular emphasis is placed on the role of BBB dysfunction as both a pathogenic mechanism and a therapeutic target, highlighting the translational opportunities and unmet clinical needs that continue to drive this rapidly evolving field.

## 2. NVU Alterations in Neurodegenerative Diseases: Current Unsuccessful Treatments and Molecular Pathways Involved Potentially to Target

**Figure 1:**
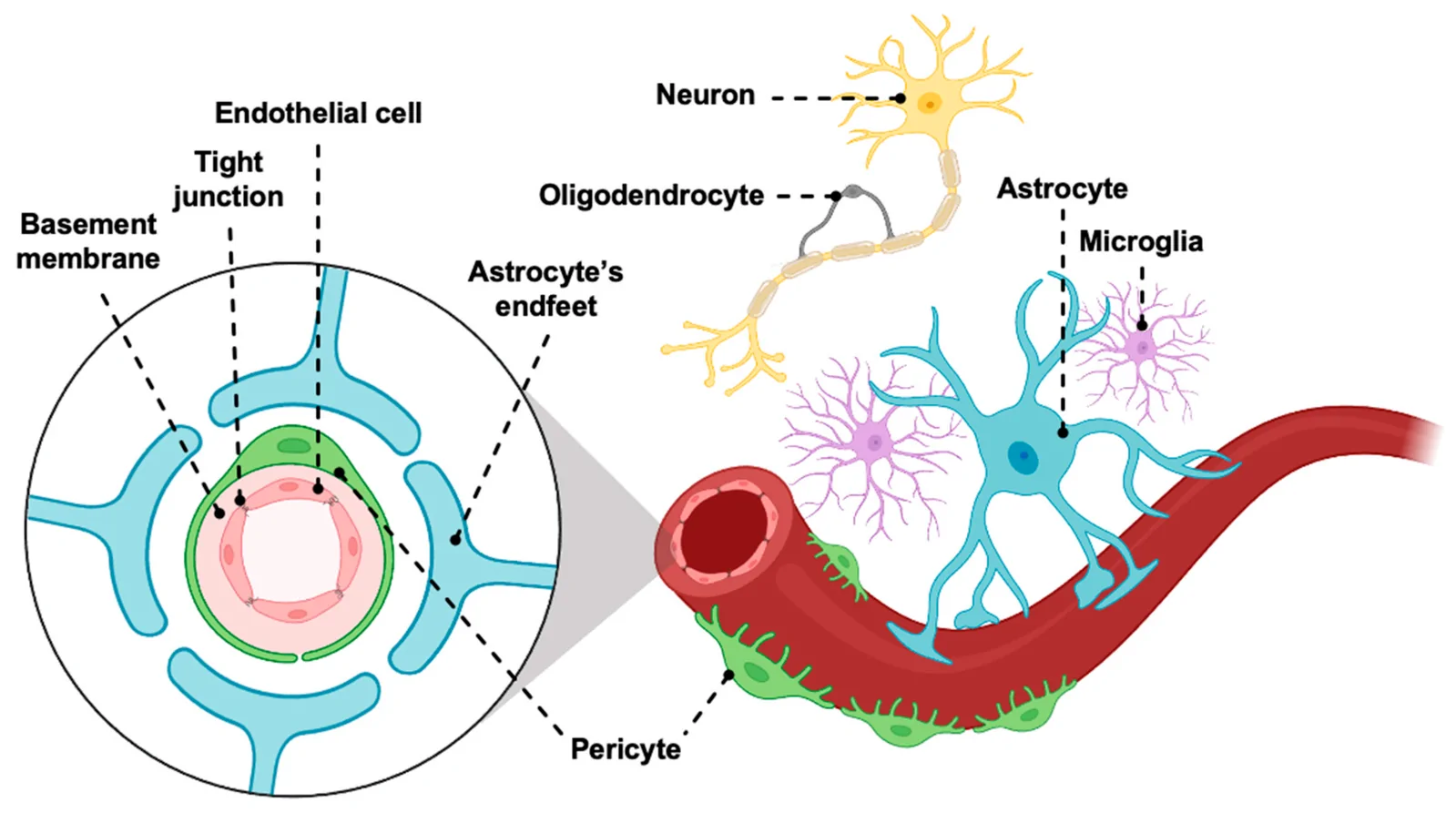
NVU structure in the physiological condition.

To fully understand how barrier dysfunction contributes to neurodegeneration and how it may be therapeutically targeted, it is necessary to consider the broader NVU, of which the BBB represents a central functional component. The NVU comprises endothelial cells, pericytes, astrocytes, microglia and neurons that operate as an integrated signaling network to coordinate cerebral blood flow (CBF), metabolic support, immune surveillance and barrier integrity [19] (Figure 1).

Far from serving as a passive structural interface, the NVU dynamically adapts to neuronal activity and environmental stimuli, thereby maintaining brain homeostasis throughout life.

Increasing evidence suggests that NVU dysfunction is not simply a downstream consequence of neuronal degeneration, but rather, an early pathogenic event that may actively drive disease initiation and progression [20,21]. BBB breakdown, pericyte degeneration, astrocytic remodeling, impaired neurovascular coupling, and chronic neuroinflammation have all been identified as early alterations in several neurodegenerative disorders, often preceding the appearance of classical neuropathological hallmarks such as amyloid plaques, neurofibrillary tangles or Lewy bodies [22]. This paradigm shift places the NVU at the center of disease pathogenesis and highlights it as a promising source of early biomarkers and therapeutic targets.

In the following sections, we examine the major NVU-associated alterations across NDs and discuss their implications for diagnosis, disease monitoring and the development of next-generation neurotheranostic strategies.

### 2.1. Alzheimer’s Disease

Alzheimer’s disease (AD) is a slowly progressive neurodegenerative condition classically characterized by the accumulation of extracellular Aβ plaques and intracellular neurofibrillary tangles composed of hyperphosphorylated tau. However, epidemiological, clinical and experimental studies have shown that, in addition to Aβ deposition, tau pathology and neuronal loss, AD is also associated with early NVU dysfunction [20,23]. Moreover, a growing body of evidence indicates NVU dysfunction is not merely a secondary consequence of protein aggregation but an early and active contributor to disease pathogenesis [20,23,24]. This concept is reflected in the “two-hit vascular hypothesis” and the broader framework of vascular contributions to cognitive impairment and dementia, which place cerebrovascular dysfunction among the earliest events driving neurodegeneration [25,26,27].

Among NVU alterations, BBB disruption represents one of the earliest detectable abnormalities, often preceding overt cognitive decline by several years. Dynamic contrast-enhanced magnetic resonance imaging (MRI) studies have demonstrated increased BBB permeability in individuals with mild cognitive impairment independently of Aβ and tau burden [28].

At the molecular level, BBB dysfunction is associated with the loss of tight junction and adherens junction proteins, including claudin-5, occludin, ZO-1 and VE-cadherin, partly driven by the upregulation of matrix metalloproteinases (MMP-2 and MMP-9) in response to Aβ oligomers and neuroinflammatory mediators [29,30]. Tight junction disruption is documented by an altered expression of the tight junction proteins claudin-5, occludin and ZO-1 in post-mortem AD brains [31] and in vivo [32,33,34] and in vitro models [35,36]. As barrier integrity deteriorates, peripheral factors such as fibrinogen, thrombin and albumin gain access to the brain parenchyma, amplifying neuroinflammation and neuronal injury. Simultaneously, endothelial dysfunction alters Aβ trafficking across the BBB through progressive downregulation of the efflux receptor LRP1 and upregulation of RAGE-mediated influx pathways, thereby promoting cerebral amyloid accumulation [37].

Indeed, endothelial cells in AD are found in a dysregulated and pro-angiogenic state, which contributes to impair the specialized transport functions of the BBB and increase its permeability. Different transcriptomics and RNA analysis studies revealed that these cells present accumulation of AD pathological proteins, failure in the HIF1α–VEGFA signaling axis, and dysregulation of angiogenesis and immune-related processes [38].

These vascular alterations are closely linked to pericyte degeneration, which is increasingly recognized as a central event in AD pathophysiology [39,40]. Post-mortem and biomarker studies consistently demonstrate reduced pericyte coverage and elevated soluble PDGFR-β levels in AD patients, suggesting ongoing vascular injury. Aβ-induced activation of cyclophilin A-dependent inflammatory pathways, particularly in APOE4 carriers, contributes to basement membrane degradation, pericyte detachment and capillary rarefaction [41]. The resulting impairment of CBF autoregulation leads to chronic hypoperfusion, neurovascular uncoupling and metabolic stress, further compromising neuronal survival. Moreover, pericyte dysfunction negatively impacts glymphatic and transvascular clearance mechanisms, reducing the elimination of Aβ and tau species and reinforcing protein aggregation [42]. These findings suggest that pericyte loss leads to changes associated with AD pathogenesis such as BBB breakdown, altered CBF and impaired Aβ clearance, supporting the hypothesis that pericyte dysfunction contributes to AD pathogenesis [43].

Astrocytes initially respond to these pathological changes through reactive astrogliosis, accumulating around amyloid deposits and promoting their clearance via ApoE-dependent pathways and lysosomal degradation mechanisms. Although initially protective, chronic astrocyte activation progressively evolves into a dysfunctional phenotype characterized by impaired glutamate uptake, altered potassium buffering, disruption of gap-junction communication, and reduced support of neuronal metabolism through the astrocyte–neuron lactate shuttle [44]. In parallel, the loss of perivascular aquaporin-4 (AQP4) polarization disrupts glymphatic fluid dynamics, impairing the clearance of interstitial solutes and facilitating the accumulation of toxic protein aggregates [45].

In early stages, disease-associated microglia promote Aβ clearance through TREM2-dependent phagocytic pathways, highlighting the importance of innate immune surveillance in limiting pathology [46,47]. However, prolonged activation drives a transition toward a chronically inflammatory state characterized by excessive production of TNF-α, IL-1β, IL-6, reactive oxygen species and complement factors. These mediators exacerbate BBB dysfunction, enhance synaptic pruning and amplify neuronal damage, creating a self-perpetuating cycle of neuroinflammation and neurodegeneration [48]. The AD-associated microglia phenotype is strictly dependent on TREM2 gene expression [46,47] and is characterized by both an altered activation state and a neurodegenerative phenotype, which result in an excessive release of proinflammatory cytokines and other key inflammatory mediators, contributing to neuronal loss [48].

Moreover, during AD progression, microglia have been found to be a key mediator in the generation of senile plaques through direct binding with the aggregated fibrils [49,50]. Recent single-cell transcriptomic studies have further revealed substantial microglial heterogeneity within AD brains, identifying distinct populations involved in plaque containment, vascular surveillance and inflammatory regulation.

At present, the approved pharmacological treatments are largely limited to symptomatic therapies, including cholinesterase inhibitors and NMDA receptor antagonists, which provide modest benefits by alleviating symptoms and slowing cognitive decline without preventing or limiting disease progression. More recently, the therapeutic landscape is expanding to include antibody-based therapeutics such as lecanemab [51] and donanemab [52], which have demonstrated significant clinical efficacy in patients with early AD. However, their benefits remain largely restricted to the early symptomatic stages of the disease, highlighting the need for therapeutic strategies that target pathogenic mechanisms occurring even earlier.

In this context, given the early involvement of NVU in AD pathogenesis, as stated before, preserving or restoring NVU integrity represents a promising complementary therapeutic approach with the potential to extend the window for intervention beyond that afforded by current treatments. Consistent with this concept, early preclinical studies aimed at restoring pericyte function have provided encouraging proof of concept for targeting NVU dysfunction [53]. Therefore, the NVU emerges not only as a valuable source of early biomarkers but also as an attractive therapeutic target for the development of neurotheranostic strategies capable of delaying or preventing disease progression before the onset of overt neurodegeneration.

### 2.2. Parkinson’s Disease

Parkinson’s disease (PD) is the second most common neurodegenerative disorder, with a highly rising prevalence [54]. It results from the gradual degeneration of dopaminergic neurons of the substantia nigra pars compacta. This process is responsible for the hallmark motor symptoms, including tremors, bradykinesia and postural instability [55]. PD can be classified as a synucleinopathy, since it is characterized by abnormal cytoplasmic protein (alpha-synuclein, α-syn) accumulation known as Lewy bodies in the brain and other organs [56].

Although traditionally considered a neuron-centered disorder, increasing evidence indicates that NVU dysfunction contributes significantly to disease initiation and progression. In particular, α-syn pathology extends beyond neurons and directly affects vascular and glial components of the NVU, establishing a complex interplay between protein aggregation, cerebrovascular dysfunction and neuroinflammation [57].

Accumulating evidence suggests that α-syn exerts direct toxic effects on cerebral endothelial cells and pericytes. Aggregated α-syn species have been detected within endothelial cells, where they impair mitochondrial function, increase reactive oxygen species production and activate NF-κB-dependent inflammatory pathways. These changes result in downregulation of tight junction proteins, including occludin and claudin-5, leading to increased BBB permeability and endothelial activation. Concurrent upregulation of adhesion molecules such as ICAM-1 and VCAM-1 promotes leukocyte adhesion and transmigration across the vascular wall, amplifying local inflammatory responses [58]. Pericytes are similarly affected by α-syn accumulation, exhibiting contractile dysfunction, reduced vascular coverage and impaired regulation of capillary blood flow. Furthermore, exosome-mediated transfer of α-syn between neurons, astrocytes, endothelial cells and pericytes has been proposed as a mechanism facilitating the propagation of pathology throughout the NVU [59].

The activation of endothelial cells is also observed in PD and could trigger the upregulation of cell-adhesion molecules such as VCAM-1 and ICAM-1, promoting invasion of peripheral immune cells [60]. Moreover, an altered expression of tight junction proteins, in particular occludin and zonula occludens-1, has been found in post-mortem brain tissues from PD patients and in hCMEC/D3 cells incubated with α-synuclein preformed fibrils caused by alpha-synuclein accumulation [61].

These alterations contribute to progressive cerebrovascular dysfunction and neurovascular uncoupling, both increasingly recognized as important features of PD. Neuroimaging and post-mortem studies have demonstrated reductions in CBF, particularly within the basal ganglia, substantia nigra and posterior cortical regions, where perfusion deficits correlate with motor and cognitive impairment. Impaired cerebrovascular reactivity reflects endothelial dysfunction and loss of pericyte-mediated vascular regulation, while reduced endothelial nitric oxide bioavailability further compromises vasodilatory responses and the ability of the cerebral vasculature to adapt to neuronal metabolic demands. The resulting mismatch between neuronal activity and blood supply may exacerbate metabolic stress and increase the vulnerability of dopaminergic neurons to degeneration [62].

The cerebrovascular alterations described in PD mainly include abnormal angiogenesis, inflammation and increased BBB permeability. Studies investigating angiogenesis in PD reported opposite results, some pointing to endothelial cell and vessel degeneration, described in the brains of PD patients and genetic models [63,64], as well as others reporting excessive angiogenesis in post-mortem brains, which is instead replicated in toxin-based animal models [65,66,67]. This indicates that both processes do not exclude each other and are actually suggested to be stage dependent, with the early stages being associated with pericyte activation and angiogenesis while later stages are linked to vascular rarefication [64].

Neuroinflammation represents another critical component of NVU dysfunction in PD [68]. Activated microglia are consistently observed in the substantia nigra of PD patients and experimental models, where they release proinflammatory cytokines, reactive oxygen species and other neurotoxic mediators that amplify neuronal injury. Importantly, growing evidence suggests that microglial priming may occur long before the onset of overt motor symptoms, creating a permissive environment for subsequent neurodegeneration [69]. These observations support the hypothesis that neurovascular and inflammatory alterations may precede classical nigrostriatal degeneration and participate in the earliest stages of disease development [70,71,72]. Microglial cells are able to produce factors involved in the pathophysiology of PD and are highly present in the perivascular space. Recent evidence suggests that perivascular microglia are able to maintain vascular integrity in normal conditions but phagocytose BBB vessels upon prolonged inflammation [73], and the number of perivascular microglia seems to be strongly associated with the level of pericytes in PD [64].

In this context, the gut–brain axis has emerged as a particularly intriguing contributor to early NVU dysfunction. Intestinal dysbiosis and increased gut permeability may facilitate the systemic dissemination of microbial products such as lipopolysaccharides, which can activate peripheral immune pathways, promote BBB dysfunction and trigger microglial activation [74].

Astrocytes also undergo profound functional changes during PD progression. Under physiological conditions, astrocytes provide essential trophic and metabolic support to dopaminergic neurons through glutathione synthesis, lactate delivery, glutamate clearance and secretion of neurotrophic factors such as glial cell line-derived neurotrophic factor (GDNF). However, astrocytic accumulation of α-syn impairs autophagic pathways, reduces neurotrophic support, disrupts glutamate homeostasis through downregulation of GLT-1 transporters and promotes the release of proinflammatory mediators, including complement component C3. These alterations contribute to excitotoxicity, chronic neuroinflammation and progressive neuronal dysfunction [75].

Pericytes are one of the first responders to systemic inflammation [76]. Upon α-synuclein exposure, pericytes undergo morphology and marker expression changes toward a reactive and migratory phenotype, which is associated with increased angiogenesis [77,78]. Direct evidence from an in vitro study demonstrated that exposure to monomeric α-syn leads to a significant release of proinflammatory molecules in pericytes that, in turn, induces hyperpermeability in endothelial cells [79].

Current therapeutic strategies for PD remain largely symptomatic, primarily targeting dopaminergic dysfunction through levodopa in combination with decarboxylase inhibitor or dopamine agonists. Although these approaches provide substantial relief of motor symptoms, their long-term efficacy is limited by the development of motor fluctuations, dyskinesias, and other treatment-related adverse effects, while failing to modify the underlying neurodegenerative process [80]. The lack of disease-modifying therapies underscores the need to identify novel pathogenic mechanisms that can be therapeutically exploited.

In this regard, the growing recognition of NVU dysfunction as an early and active component of PD pathophysiology provides a compelling rationale for exploring neurovascular-targeted interventions. Beyond contributing to disease progression, BBB dysfunction may also influence drug delivery and therapeutic efficacy by altering transport mechanisms and brain permeability. Despite these observations, no therapeutic strategies specifically aimed at preserving or restoring BBB integrity have yet been evaluated in clinical trials for PD.

Nevertheless, emerging preclinical evidence supports the feasibility of targeting NVU. Transplantation of induced pluripotent stem cell (iPSC)-derived pericytes has been proposed as an approach to promote BBB regeneration and restore vascular integrity [81]. Supporting this evidence, there is a study showing that in an experimental murine stroke model, transplanted iPSC-derived pericytes were able to successfully improve BBB integrity and promote neurological recovery [82]. In another work, downregulation of vascular protein expression of P-glycoprotein was reversed by vitamin D receptor ligand-1α,25-dihydroxyvitamin D3 (1,25(OH)2D3) treatment in a 6-hydroxydopamine (6-OHDA)-induced PD mouse model and in a combinatorial AAV–α-syn/α-syn preformed fibril (PFF) in vitro model [83], suggesting a potential target for the maintenance of vascular homeostasis in PD pathology. Although these approaches remain at the preclinical stage, they support the concept that preserving NVU function and BBB integrity could complement conventional dopaminergic therapies and represent a promising avenue for the development of future disease-modifying strategies in PD.

### 2.3. Amyotrophic Lateral Sclerosis

Amyotrophic lateral sclerosis (ALS) is a progressive neurodegenerative disorder characterized by the selective loss of upper and lower motor neurons, leading to progressive muscle weakness, paralysis, and ultimately, respiratory failure [84]. To this day, there are no validated biomarkers for this disease other than neurofilament accumulation, a feature shared with other neurodegenerative disorders.

Although traditionally considered a motor neuron-centered disease, increasing evidence indicates that NVU dysfunction contributes substantially to disease initiation and progression. Alterations of the BBB have been consistently documented in both patients and experimental models, suggesting that vascular pathology may represent an early and integral component of ALS pathogenesis.

Dysfunctions in the permeability of the BBB and blood–spinal cord barrier (BSCB) are present in ALS patients with both the familial and sporadic form [43]. Over twenty independent studies in post-mortem human tissue and in CSF sampling from living ALS patients have reported the presence of erythrocyte extravasation and reductions in pericyte population [85]. At a molecular level, transcriptional analysis has demonstrated a reduction in the tight junction proteins of the BSCB associated with elevations in MMP-9 [86].

Among the earliest detectable abnormalities are structural and functional alterations of the BBB and BSCB. Reduced expression of tight junction proteins including claudin-5, occludin and ZO-1, together with pericyte degeneration and basement membrane remodeling, have been observed before significant motor neuron loss in several ALS models [87]. Reduction in capillary diameter and density is associated with a reduced expression of the adhesion molecule PCAM-1, suggesting early damage to endothelial cells. The same study revealed a significant and progressive decrease in tight junction components, including occludin, from large motor neurons and endothelium. Moreover, increased MMP-9 activity in the cortex and spinal cord of post-mortem patients is replicated in the large motor neurons of ALS mice, progressively appearing in blood vessel-like structures and microglia. The changes observed in this transgenic mouse model lead to spontaneous BSCB disruption, and this process is probably induced by accumulation of blood-derived neurotoxic hemoglobin and iron in the spinal cord [88]. Treatment targeting neurotoxic hemoglobin and iron deposits is able to delay the onset of motor-neuron impairment and degeneration, suggesting that restoring BSCB integrity during an early disease phase retards the disease process.

Evidence of microhemorrhages, hemosiderin deposits and erythrocyte extravasation further supports the presence of early barrier dysfunction. As vascular integrity deteriorates, circulating proteins, complement components, inflammatory mediators and oxidative stress-inducing molecules gain access to the neural parenchyma, creating an environment that increases motor neuron vulnerability [89].

The central role of NVU dysfunction is further highlighted by the involvement of TAR DNA-binding protein 43 (TDP-43) and fused in sarcoma (FUS), the major pathological proteins associated with ALS. Although these proteinopathies have traditionally been studied in motor neurons, pathological TDP-43 and FUS accumulation has also been identified in endothelial cells and astrocytes, indicating that NVU components are active participants in disease progression. In endothelial cells, TDP-43 dysfunction alters the expression of genes involved in angiogenesis, vascular homeostasis and barrier maintenance, exacerbating BBB and BSCB disruption [90,91]. Simultaneously, protein aggregation within astrocytes compromises multiple homeostatic functions that are essential for motor neuron survival.

Astrocytic dysfunction is particularly prominent in ALS and represents one of the clearest examples of non-cell-autonomous neurodegeneration. Patient-derived astrocytes acquire a toxic phenotype characterized by both loss of neuroprotective functions and gain of neurotoxic properties [92]. Experimental studies have demonstrated that conditioned media from ALS astrocytes selectively induce motor neuron death, suggesting the release of soluble toxic mediators, including reactive oxygen species, aberrant lipid metabolites, inflammatory cytokines and mutant protein species in familial forms of the disease [93]. In parallel, downregulation of the glutamate transporter GLT-1 (EAAT2) impairs synaptic glutamate clearance, promoting chronic excitotoxicity [94]. Given the high expression of calcium-permeable AMPA receptors in motor neurons, this excitotoxic environment is particularly detrimental and contributes directly to neuronal degeneration [95]. Furthermore, reduced secretion of trophic factors such as VEGF and IGF-1 further compromises neuronal resilience.

Neuroinflammation provides an additional layer of NVU dysfunctions. Microglia undergo dynamic phenotypic changes throughout disease progression, transitioning from relatively protective states during early stages to chronically activated, proinflammatory phenotypes in advanced disease. Activated microglia release cytokines, reactive oxygen species and complement factors that amplify tissue injury and exacerbate motor neuron loss. Importantly, disruption of the BSCB facilitates the infiltration of peripheral immune cells, including cytotoxic T lymphocytes and regulatory T cells, introducing an adaptive immune component to ALS pathology [96]. This complex interaction between vascular dysfunction, glial activation and peripheral immunity suggests that the immune–vascular interface plays a critical role in disease progression. Indeed, microglial cells are considered as a double-edged sword, supporting the importance of targeting microglia to minimize cytotoxicity and maximize neuroprotection in NDs [97].

Current therapeutic options for ALS remain limited and are not curative for the disease. Before 2024, only two pharmacological agents had been approved by the U.S. Food and Drug Administration (FDA) and were available in the United States: the glutamate antagonist Riluzole and the antioxidant Edaravone. However, these compounds exert only a moderate effect on disease progression, increasing survival by two to six months [84]. The development of new disease-modifying therapies based on antisense oligonucleotides may have a beneficial effect in ALS patients with specific gene mutations. In 2023, the FDA approved the first therapy with an antisense oligonucleotide (Tofersen). The use of Tofersen represents a major advancement in ALS therapy for SOD1 mutation carriers, given that the ALS population with mutant SOD1 accounts for 15–20% of familiar ALS and approximately 3% of sporadic ALS [98].

A promising therapeutic option to treat endothelial cell-based neurological diseases was proposed by Reijerkerk et al., who were able to identify a set of microRNAs modulating BBB function under normal and inflammatory conditions [99]. Among them, miR-125a-5p was shown to be a key regulator of brain endothelial tightness, suggesting that its administration could potentially strengthen BBB function in multiple sclerosis. The identification of other BBB-associated miRNAs may enable the development of novel therapeutic strategies aimed at restoring brain vascular function across different NDs, including ALS.

### 2.4. Huntington’s Disease

Huntington’s disease (HD) is an autosomal-dominant neurodegenerative disorder caused by an expanded CAG trinucleotide repeat in the huntingtin (HTT) gene, leading to the production of mutant huntingtin (mHTT) protein [100]. Although HD has traditionally been considered a neuron-centered disease characterized by progressive degeneration of striatal medium spiny neurons, increasing evidence suggests that NVU dysfunction contributes significantly to disease pathogenesis [101].

mHTT is widely expressed throughout the NVU, including in endothelial cells, pericytes, astrocytes and microglia. In cerebral endothelial cells, mHTT disrupts mitochondrial homeostasis through altered mitochondrial dynamics, increased Drp1-mediated mitochondrial fission, elevated reactive oxygen species production and reduced nitric oxide bioavailability [102]. These alterations impair endothelial function, compromise vasodilatory responses and reduce CBF, particularly within the striatum, a region characterized by exceptionally high metabolic demands and selective vulnerability to neurodegeneration. Pericytes are similarly affected by mHTT-associated cellular stress, further contributing to vascular instability and impaired regulation of microvascular perfusion.

One of the most striking manifestations of NVU dysfunction in HD is the presence of profound cerebral metabolic abnormalities that can be detected years before the onset of clinical symptoms [103]. Positron emission tomography studies consistently demonstrate reduced glucose utilization in presymptomatic mutation carriers, indicating that metabolic impairment is an early event in disease progression [104]. This energetic deficit is exacerbated by endothelial dysfunction, particularly through reduced expression of the glucose transporter GLUT1 at the BBB, which limits glucose delivery to neurons already burdened by mHTT-induced mitochondrial dysfunction [105]. Concurrently, abnormalities in neurovascular coupling impair the ability of local blood flow to adapt to neuronal activity, restricting activity-dependent delivery of oxygen and metabolic substrates. Functional MRI studies have revealed alterations in blood oxygen level-dependent responses consistent with disrupted neurovascular communication, supporting the concept that vascular dysfunction contributes directly to neuronal energy failure.

Astrocytes and microglia further amplify these pathogenic processes. Astrocytic expression of mHTT impairs multiple homeostatic functions, including glutamate clearance through downregulation of GLT-1 transporters, thereby increasing extracellular glutamate concentrations and promoting excitotoxic stress within vulnerable striatal circuits. In parallel, astrocytic metabolic dysfunction reduces support for neuronal energy demands and may further compromise neurovascular regulation. Microglia also exhibit early activation in HD, initially participating in debris clearance and tissue surveillance but progressively adopting a chronic proinflammatory phenotype that contributes to neuronal injury. Notably, neuroinflammatory changes can be detected during prodromal stages of disease, with TSPO-PET studies demonstrating microglial activation in asymptomatic mutation carriers many years before expected clinical onset [106].

Collectively, these findings support a model in which mHTT-driven dysfunction of vascular, glial and immune components of the NVU contributes to the establishment of a chronic state of metabolic insufficiency, impaired neurovascular communication, and neuroinflammation. Importantly, many of these alterations arise long before overt neuronal loss becomes clinically apparent, highlighting the NVU as a potential source of early biomarkers and therapeutic targets. The early detectability of metabolic and inflammatory changes through advanced imaging approaches further underscores the potential of neurotheranostic strategies aimed at identifying vulnerable individuals during the prodromal phase and delivering targeted interventions before irreversible neurodegeneration occurs.

### 2.5. Physiological and Pathological Aging

Aging is the strongest risk factor for NDs; however, the biological vulnerability associated with aging does not arise abruptly in late life. Rather, it reflects the cumulative impact of genetic predisposition, environmental exposures, metabolic stressors and inflammatory events acting across the lifespan [107]. Increasing evidence suggests that many of these factors converge on the NVU, progressively eroding its resilience long before overt neurodegeneration becomes clinically apparent [108].

At the vascular level, aging is associated with progressive rarefaction of the cerebral microvasculature, characterized by reduced capillary density, diminished branching complexity and increased vessel tortuosity [109]. These alterations impair tissue perfusion, promote regional hypoxia and reduce the efficiency of nutrient and oxygen delivery. Simultaneously, thickening of the vascular basement membrane due to the accumulation of collagen IV, laminin, fibronectin and advanced glycation end products increases vascular stiffness and compromises exchange between blood and neural tissue [110]. These structural changes are accompanied by the appearance of white matter hyperintensities and enlargement of perivascular spaces, neuroimaging hallmarks that reflect chronic microvascular dysfunction and impaired interstitial fluid clearance [111]. Importantly, both features are strongly associated with cognitive decline and increased risk of dementia [112].

Aging also profoundly affects endothelial biology. Brain endothelial cells progressively acquire features of cellular senescence, including activation of p16 and p21 signaling pathways and the development of a senescence-associated secretory phenotype. Through the release of proinflammatory cytokines, chemokines, matrix-remodeling enzymes and coagulation-related factors, senescent endothelial cells contribute to the chronic low-grade inflammatory state commonly referred to as inflammaging [109]. In parallel, age-related oxidative stress promotes endothelial nitric oxide synthase (eNOS) uncoupling, reducing nitric oxide bioavailability and impairing vasodilatory responses. Mitochondrial dysfunction, defective mitophagy and excessive reactive oxygen species production further exacerbate endothelial impairment, ultimately contributing to BBB breakdown, neurovascular uncoupling and chronic cerebrovascular insufficiency [113].

Pericytes undergo similarly profound age-dependent alterations. Progressive loss of pericyte coverage weakens BBB integrity and compromises the regulation of CBF. Moreover, aged pericytes exhibit reduced responsiveness to PDGF-BB signaling and increasingly adopt a fibrogenic phenotype characterized by enhanced TGF-β signaling and the expression of contractile proteins such as α-smooth muscle actin [114]. This transition promotes perivascular fibrosis, vascular stiffening and further deterioration of neurovascular coupling. Defects in calcium signaling additionally impair the ability of pericytes to dynamically regulate capillary tone in response to neuronal activity, contributing to the mismatch between metabolic demand and vascular supply that characterizes the aging brain [115].

Glial aging represents another major determinant of NVU dysfunction. Astrocytes progressively transition toward a reactive phenotype associated with loss of key homeostatic functions, including glutamate clearance, potassium buffering, metabolic support and maintenance of water homeostasis. Particularly relevant is the age-dependent loss of AQP4 polarization at astrocytic endfeet, which compromises communication between the vascular and interstitial compartments. Aging astrocytes also accumulate intracellular lipid droplets, reflecting alterations in lipid metabolism that are associated with oxidative stress, impaired trophic support and a proinflammatory transcriptional profile [116]. Concurrently, microglia undergo a shift from efficient immune surveillance toward a state of functional exhaustion and chronic priming. Senescent microglia exhibit impaired phagocytic activity, lysosomal dysfunction and increased activation of inflammasome pathways, resulting in a persistent proinflammatory environment that lowers the threshold for pathological responses to subsequent insults [117].

One of the most important consequences of these age-related changes is the progressive deterioration of glymphatic function. Efficient glymphatic clearance depends on coordinated interactions between vascular pulsatility, astrocytic AQP4 polarization and perivascular fluid dynamics [118]. Aging disrupts each of these components through vascular stiffening, sleep fragmentation, loss of AQP4 polarity and increased interstitial protein accumulation. As a result, the clearance of metabolic waste products becomes progressively less efficient, favoring the accumulation of amyloid-β, tau, α-synuclein and other aggregation-prone proteins [119]. Notably, genetic risk factors such as APOE4 further impair glymphatic function, suggesting that inherited susceptibility and biological aging converge on common NVU-dependent pathways.

These findings support the concept that aging drives a progressive, multisystem deterioration of the NVU involving vascular, glial, immune and clearance mechanisms. Rather than representing isolated alterations, these processes interact to create a self-reinforcing cycle of inflammation, metabolic insufficiency, barrier dysfunction and impaired waste clearance that lowers the threshold for neurodegenerative disease [108]. From a neurotheranostic perspective, many of these age-associated changes are detectable before overt neurodegeneration occurs, through advanced imaging, fluid biomarkers and molecular profiling approaches [113]. Consequently, the aging NVU represents not only a critical target for preventive interventions but also a promising platform for the development of integrated diagnostic and therapeutic strategies aimed at preserving brain resilience across the lifespan.

From a life-course perspective, the aging NVU can be viewed as the cumulative product of processes initiated during earlier stages of life. Environmental exposures occurring during neurodevelopment may alter barrier maturation, vascular architecture, immune programming and metabolic homeostasis, thereby establishing latent vulnerabilities that remain clinically silent for decades [120]. During aging, these vulnerabilities may become progressively unmasked through endothelial senescence, pericyte loss, glial dysfunction and impaired waste-clearance mechanisms, ultimately lowering the threshold for neurodegenerative disease.

### 2.6. Cross-Disease Comparison and Theranostic Implications

This cross-disease analysis reveals a convergent NVU pathological signature shared across neurodegenerative conditions despite their distinct molecular triggers. A comparative overview of the different NVU components in physiological conditions across NDs and aging is provided in Table 1 and Table 2, respectively, facilitating the identification of common pathological signatures and disease-specific patterns. This convergence has profound theranostic implications: NVU-targeted platforms, whether imaging agents, nanocarriers or immunomodulatory strategies designed around shared pathological endpoints (BBB disruption, pericyte loss, neuroinflammation) may offer transdiagnostic utility, reducing development costs and accelerating clinical translation. Conversely, disease-specific molecular features—Aβ/tau in AD, α-syn in PD, TDP-43 in ALS, mHTT in HD—can serve as molecular barcodes for targeted theranostic personalization, enabling precise discrimination between conditions that share overlapping NVU phenotypes.

## 3. Role of miRNAs and Their Utilities as Diagnostic/Prognostic Biomarkers in Precision Medicine

An increasing number of studies is available reporting evidence that miRNAs have a role as key regulators of genes with important functions in the regulation of networks in NVU cells.

They could be carried by extracellular vesicles (EVs) as well as by exosomes (EXs) acting as modifiers on the structure and function of the NVU [122,123]. miRNAs allow communication among the different NVU parts, regulate the production of proteins important in the BBB stability, and finally, could affect several processes known to be essential keys in neurodegeneration such as apoptosis, neuroinflammation, and glial cell excitation [124].

Different NVU cells express different miRNAs, each involved in different processes. We can group miRNAs associated with endogenous NVU regulation/alteration as follows: miRNAs involved in the modulation of immune cell activation (e.g., miR-9, miR-124, and miR-155); miRNAs involved in the regulation of neuroinflammation (e.g., miR-9, miR-21, miR-146, miR-155); miRNAs involved in the regulation of NVU integrity and angiogenesis (e.g., miR-132, miR-124, and miR-29); miRNAs involved in the regulation of BBB integrity (e.g., miR-124, miR-155, and miR-146a).

Moreover, for a few numbers of miRNAs, involvement in several processes related to NVU and possibly to neurodegeneration was reported. The miR-27a-3p, for example, was reported to increase the expression of occludin and claudin-5, thus promoting the stability of the BBB [125]; the let-7 family, the miR-23/27/24 cluster, and miR-1290 stimulate endothelial cell proliferation and neovascularization, promoting the repair of damaged blood vessels [126]; miR-21 and miR-124 are involved in regulating cell survival, providing neuroprotection by targeting crucial apoptosis-related genes like B-cell lymphoma 2 (Bcl-2) and different members of the caspase gene family; miR-21-5p promotes the integrity of the BBB by downregulating proinflammatory cytokines such as TNF-α and IL-6, thus decreasing inflammation [127].

Considering the role of NVU in neurodegenerative disorders, it is worth noticing that several miRNAs involved in BBB maintenance and stability and in the regulation of vascular tension and inflammatory responses have been reported to change with age. These age-associated miRNA shifts may affect gene networks linked to neurodegenerative risk and cognitive decline [128]. One among these age-dependent miRNAs is miR-34a, which was studied in AD in connection with pathways involved in the regulation of activity-dependent plasticity, memory decline and neurodegeneration, and myelin-associated adaptation [129]. Questioning the mechanisms through which the expression of such a miRNA could be modified during aging, it was hypothesized and confirmed that a possible explanation could be related to a regulation by stress-responsive transcription factors at its promoter level. The miR-34a promoter has, in fact, been studied, and it was found to contain binding sites for p53, NF-κB, and STAT3, all of which can become activated in aging tissues under conditions of oxidative stress and chronic low-grade inflammation [130]. In particular, the p53/miR-34a/SIRT1 axis provides a possible framework linking DNA damage, senescence-associated signaling, and metabolic adaptation. A gap in knowledge persists due to the fact that even if a reason for the miRNA age-dependent regulation has been found, this discovery does not allow us to explain the observed tissue- and cell-specific changes.

An increased interest in studying changes in miRNAs’ expression, with the aim of identifying candidate biomarkers of aging, have been reported. However, many of them did not show causal relationships, but only associations, with a strength of evidence that varies across each couple comprising the miRNA and proposed target [131].

The advantage of studying miRNAs as candidate biomarkers across many disease settings, and in particular when biopsies are undesirable, comes from the observation that they are relatively stable in biological fluids, being frequently associated with lipoproteins, ribonucleoprotein complexes, or EVs such as EXs, which can shield them from RNase degradation [132]. The actual limits of their uses, however, reside in the observation that only a few reported associations are reproducible due to populations’ variability, disease heterogeneity, and laboratory workflows not always being standardized.

Nevertheless, miRNAs are widely used as biomarkers in NDs, mostly AD and PD.

In the case of AD, a set of miRNAs (miR-26b, miR-30e, miR-34a, miR-34c, miR-107, miR-125b, miR-146a, miR-151, miR-200c, miR-210, and hsa-miR-485) is commonly used and suggested to predict the onset of the disease 20 years before the first clinical signs [133]. A separate study identified five miRNAs (miRNA-9, miRNA-34a, miRNA-125b, miRNA-146a, and miRNA-155) that were abundant in AD brain tissue and that contributed to the pathophysiology of the disease, being involved in the regulation of inflammatory pathways and classified as proinflammatory miRNAs [133]. Among the above miRNAs, some cause the modulation of different neurotropic factors, and hence, affect the NVU integrity to control neurovasculature function. Examples of this kind of miRNAs are miR-26b, found to directly regulate BDNF activity and expression, and miR30e, which modulates NLRP3 inflammation and has a potential role in reducing BDNF production in AD patients. Other miRNAs, such as miR-210 and miR-107, act by disrupting junction proteins (i.e., occludin, ZO-1 and β-catenin), hence damaging the BBB’s integrity. A similar effect was reported for miR-34a, whose dysregulation in cerebrovascular cells increases BBB permeability and triggers endothelial cell injury. Moreover, in astrocytes, miR-210 regulates apoptosis and angiogenesis, showing also anti-inflammatory effects. MiR-155 is a significant contributor to neuroinflammation due to its functions in the regulation of cytokine production via macrophage coordination. MiR-146a, mainly expressed in astrocytes and microglia, is considered a crucial regulator involved in several processes linked to AD: it has a role in regulating NF-kB, and it could attenuate astrocytic inflammation; it plays a role in inducing TLR tolerance in macrophages; and finally, it is involved in microglial polarization [134].

MiR-485-5p was reported to have a suppressive role in AD progression, probably due to its role in targeting PACS1, thus inhibiting the apoptosis of pericytes, which represent key cellular components of the NVU that maintain BBB integrity. Some miRNAs were studied due to their ability in regulating Aβ and tau expression. For example, miR-339-5p was demonstrated to be capable of downregulating the expression of β-site APP-cleaving enzyme 1 (BACE1), an enzyme with a crucial role in cleaving the Aβ precursor into insoluble Aβ filaments. This miRNA is, then, a hot targeting candidate to be used in precision medicine therapy, considering that restoring miR-339-5p levels in AD patients might slow down the Aβ filaments’ deposition and accumulation, reducing the cognitive impairment progression [135]. Another miRNA with a role in regulating protein accumulation is miR-124-3p, which was demonstrated to attenuate the apoptosis of neuroblastoma cells in vitro by inhibiting the abnormal hyperphosphorylation of tau protein and targeting caveolin-1 [136]. For a comprehensive review of miRNAs’ role in AD, please read [137].

In the case of PD, the most relevant and studied miRNAs are miR-30, miR-29, let-7, miR-485 and miR-26, which were found involved in the pathogenesis of the disease [138]. Moreover, circulating miRNAs could be detected in biological fluids (blood, CSF, serum, saliva, urine), and they are seen as mediators of cellular communication. A great effort has been made in verifying if these circulating miRNAs could be useful in ND diagnosis as biomarkers as well as regulators. For instance, let-7, miR-128, miR-433, miR-485-5p, miR-132, and miR-212 were all found to be dysregulated in the CSF of PD patients, suggesting a possible use as biomarkers and potential candidate targets for therapy [139]. In PD, high levels of miR-132-3p suppress the protective protein GLRX (glutaredoxin-1). This loss causes microglia to over-activate and release harmful cytokines. These chemicals create a toxic loop that damages brain cells. Studies in cellular models showed that this observation might be useful for a potential therapeutic strategy: the stimulation of GLRX is able to overpower the miRNA toxic effects and, in turn, it may suppress the dopaminergic neuronal damage and neuroinflammation [140]. Additionally, miR-124 modulates a number of pathways involved in PD pathogenesis and NVU physiology and functioning (NF-κB, STAT3, calpain 1/p25/cyclin-dependent kinases 5 (CDK5), ERK-mediated, 5′ adenosine monophosphate-activated protein kinase (AMPK), and Bcl-2-interacting mediator of cell death (Bim)) that regulate cell survival, apoptosis, autophagy, neuroinflammation, oxidative damage and mitochondrial dysfunction [141]. Considering the implication of the latter mentioned miRNA in PD pathogenesis even at initial stages, it was speculated that it could be a candidate preclinical biomarker at pre-symptomatic stages and, due to its demonstrated neuroprotective effect, it has been studied as a potential miRNA to be used in therapy, hypothesizing that an increase in its intracellular levels by external targeted delivery could reduce cell damage [141]. Other candidate miRNAs suggested as potential biomarkers and therapeutic targets relevant to NVU are miR-26a, which could be upregulated by a therapeutic strategy aimed to downregulate the death-associated protein kinase 1 (DAPK1), a widely distributed serine/threonine kinase that is positively correlated with neuronal synuclein in PD [142]; miR-15a-5p, which was reported to be a target of LncRNA rhabdomyosarcoma 2-associated transcript (RMST), which is highly expressed in the serum of PD patients and is considered a potential biomarker in the disease diagnosis. The RMST serves as a key regulator of neural stem cells to regulate the release of inflammatory cytokines and neuronal apoptosis [143].

LncRNAs are implicated in both the dysregulation of motor neuron survival pathways in ALS and in the aggregation of mHTT proteins in HD [144].

The use of non-coding RNAs in precision medicine as tools of therapy is challenging due to some limitations. One of them is the way to administer exogenous miRNAs. Some advancement in delivery systems occurred with the use of viral vectors, which, however, do not overcome some issues such as the miRNAs’ degradation in the circulation and low efficiency of penetration to the brain, and more recently, with the use of natural EXs originating from the multivesicular endosomal cell compartment. Distinct profiles of exosomal miRNAs were reported in neurodegenerative disorders, suggesting exosomal miRNAs may serve as potential biomarkers for diagnosing CNS diseases [145]. The advantages of EXs as vehicles to target specific therapeutic miRNAs over existing miRNA delivery vehicles reside in the fact that they are derived directly from the patient’s own cells, so they would be less immunogenic, and they remain relatively stable in the blood, as they avoid coagulation factors and complement, most likely due to their surface expression of CD46 and CD59 [146]. EXs are able to cross the BBB, but the efficiency of targeting in the brain is challenging because they usually localize in the spleen and liver. Engineered EXs targeting specifically the brain were also produced.

The main challenges in the use of miRNAs as therapeutic drugs are to avoid cell damage caused by altered toxicity, immune activation, or off-target effects and, at the same time, to improve their affinity potency. This is very important, especially in the CNS, where limited tissue accessibility and long-term exposure complicate translational design.

## 4. Discussion, Conclusions and Future Direction: Precision Medicine-Driven Strategies

While there are several definitions for theranostics (often referred to as a “see what you treat, treat what you see” technique), the concept has been applied in oncology to image and treat cancer patients for years, noted Phillip Kuo, MD, PhD, director of theranostics for the City of Hope in Duarte, CA. However, providing a recent new definition, Kuo said the technique translates to brain disease: “A theranostics system integrates some form of diagnostic testing to determine the presence of a molecular target for which a specific drug is intended” [147]. Brain disorders are challenging to diagnose and treat because the BBB restricts the delivery of therapeutic and diagnostic agents to the brain. Moreover, repeated imaging to monitor disease progression and treatment response is time-consuming and burdensome. Theranostic strategies, based on the co-delivery of imaging and therapeutic agents, provide a more efficient approach by enabling simultaneous diagnosis and treatment. Nanotechnology has emerged as a promising platform for theranostics, as nanocarriers improve targeted delivery across the BBB, enhance drug bioavailability, reduce off-target effects, and enable the combined transport of therapeutic and imaging agents, improving the management of brain disorders [148].

To date, there are no fully FDA-approved nanoparticle-based theranostic agents for brain diseases, as current nano-approvals target systemic cancers or other non-brain uses. However, several promising candidates are advancing through clinical trials or hold special regulatory status.

Lipid nanoparticles (LNPs) show great promise for brain tumor treatment and imaging by targeting cells and crossing the blood–brain barrier, but they face major hurdles like delivery barriers and tumor diversity. Better designs help LNPs cross the blood–brain barrier and reach tumor cells; stimuli-responsive LNPs release drugs using local cues like pH or enzymes, delivering multiple drugs together, thus helping stop drug resistance. Moreover, these treatments can match a patient’s specific tumor biomarkers as well, opening possibilities of advanced imaging and tracking drug delivery as it happens. Unfortunately, all these issues still face a plethora of current challenges, especially barrier crossing. Indeed, many LNPs still struggle to reach high enough levels in the brain, and off-target buildup can cause unwanted systemic toxicity. Lastly, brain tumors and brain diseases differ greatly from patient to patient, making universal designs hard to use [149].

Recently, a non-metallic MRI contrast agent called TARGET has been developed to solve two major glioma problems: mapping tumor edges before surgery and telling true growth from fake healing signs. It uses special lipid nanoparticles and engineered mRNA to light up water channels in cancer cells while fading them in healthy tissue [150].

Glioblastoma is an aggressive brain tumor with a low median survival of 12–18 months. Standard therapies fail because the blood–brain barrier blocks drugs from reaching the tumor. New treatments, such as targeted super-selective arterial delivery and ultrasound, aim to safely bypass this barrier and improve patient outcomes [151].

A recent theranostic strategy in the field of neurodegeneration relies on aptamers, single-stranded DNA or RNA molecules that bind to specific biological targets like antibodies. When paired with carbon nanotubes, these combined structures create powerful hybrid tools for medical use, improving disease detection, imaging, and targeted drug delivery [152].

In light of the needs for precision medicine and tailored theranostic approaches, in the confines of this review, we aimed to expound upon the nano-based theranostic approaches employed in the context of neurological and brain disorders.

It is clearly evident that the role of NVU has become important to every single nano-theranostic strategy. Indeed, the specific NVU-associated changes occurring during disease onset and progression, together with the natural aging process, make barriers hard to overcome, thus affecting the theranostic strategy. Nanomedicine is providing continuous innovation and new tools for overcoming barriers, though the development process is often abandoned during the preclinical studies. The reason for these failures needs further efforts directed to understand, from the early phase of disease, the intrinsic role of the NVU and brain milieu. This is clearly, dramatically evident during brain tumor disease treatments, making the associated drug resistance, and escaping drug targeting, the principal reasons for malignancy progression toward fatal outcomes. Nevertheless, the same issues are encountered in a plethora of NDs along which the NVU-associated functional changes occur. It is a sort of chicken-and-egg, well-known matter: brain diseases develop first as a consequence of NVU dysfunction, or NVU dysfunction, by itself, makes a null first step for disease onset and progression. In light of this evidence, it becomes mandatory to identify early the specific NVU changes occurring in patients suffering from brain diseases, tailoring the nanomedicine-based theranostics, as much as possible, to be adherent to the patient’s current NVU functions. It is time we stop considering the NVU as simply providing barrier functions; indeed, the huge and deep interconnections among the brain’s microvascular endothelial cells, astrocytes, neurons, and microglia open new perspectives in the field of theranostics for the brain. Indeed, because the NVU tightly controls the passage of molecules through the BBB, it dictates how diagnostic imaging agents and therapeutics reach the brain. Identifying biomarkers for conditions like Alzheimer’s, Parkinson’s, or brain tumors requires sensitive tracers, which must first bypass the NVU. To bridge this gap, modern nanomedicine and theranostic agents need to be specifically designed to interact with the NVU first. Beyond acting as a mere pathway, the NVU is also a therapeutic target. In diseases like ischemic stroke or neurodegeneration, the NVU breaks down, leading to neuroinflammation and cell death. Theranostics in this context aims to use dual-action agents: diagnose the extent of NVU and BBB breakdown using MRI or PET imaging and deliver neuroprotective drugs or targeted cellular therapies to restore tight junctions, calm glial responses (microglia and astrocytes), and promote neuronal survival. We need to consider that all these approaches are prone to failure if we are missing the precision patient-driven tailoring of the therapy. In light of these obstacles to a successful theranostic approach, we definitely need to change our perspectives: it will remain of limited significance to have one pill for one disease, providing no further improvement in the population affected by it, and becomes, indeed, the only solution to aim at one pill for patients affected by disease. The perspective becomes completely different shedding a light on the intrinsic relationship between early diagnosis and early treatment for every single patient rather than for a single disease.

Epigenetic regulation has emerged as a promising therapeutic target for neurological disorders, with RNA interference (RNAi) representing a key mechanism controlling gene expression and protein translation [153,154]. Several miRNAs regulate pathogenic pathways in NDs; however, their clinical application is limited by inefficient brain delivery due to both their physicochemical properties and the restrictive nature of the BBB [155,156].

Nanoparticles (0.1–100 nm) have been extensively investigated as carriers for CNS drug delivery because of their small size, large surface area, and tunable optical, thermal, and magnetic properties. Beyond improving drug transport across the BBB, they also support neuroimaging and early disease diagnosis [157]. Furthermore, targeted delivery of nucleic acid therapeutics to cerebral endothelial cells may simultaneously modulate multiple molecular pathways while preserving or restoring BBB integrity, potentially overcoming limitations associated with conventional brain-targeted biopharmaceuticals [158]. Recently, the potential of extracellular vesicle microRNA has been explored for the diagnosis and monitoring of cancers, neurodegenerative disorders, and metabolic diseases. This theranostic strategy still faces major hurdles in clinical translation. Studies show weak biological mechanisms, high EV heterogeneity, and lack of robust signatures. Overcoming these barriers requires proving exact cell communication pathways, using multi-marker panels, and applying single-vesicle analysis tools validated by large clinical studies [159].

Recent advances in nanotechnology have also enabled the development of MRI-guided magnetic micro-/nanorobots (MNRs) as an emerging theranostic platform for neuromedicine [160]. MNRs, composed of magnetic or magnetically responsive materials, can be precisely actuated by external magnetic fields to navigate complex neural and vascular microenvironments while delivering therapeutic and diagnostic payloads [161]. Importantly, MRI has evolved from a purely diagnostic modality into an integrated imaging and navigation system, enabling real-time tracking, closed-loop control, and targeted delivery of MNRs [162]. The continued development of MRI-guided MNRs relies on interdisciplinary advances in materials science, biomedical imaging, artificial intelligence (AI), robotics, and immunology, offering promising strategies to improve the diagnosis and treatment of neurological disorders [161,162,163].

One can argue that these approaches will dramatically increase health costs. This should be true only in part and mainly related to a disorganized research strategy. We are facing one of the biggest improvements for systematic reorganization of our research activities. Despite massive investment and the potential to improve neuroimaging, AI has seen low clinical uptake. Key barriers include poor research validation for real-world hospital use, a lack of meaningful clinical questions, an inability to explain AI decisions, complex operational and ethical hurdles, and unclear motivations focused on profit rather than patients [164]. Nevertheless, robust evidence supports that AI is opening a new era, and the opportunity is now real to maximize the precision medicine-oriented strategies. AI advances in computing, radiochemistry, and instrumentation now drive a major revival in theranostics. These tools improve image quality, workflow, and lesion analysis. They also enable precise predictive dosimetry and smarter drug design, though several clinical and technical challenges remain [165]. The convergence of nanomedicine and AI is fundamentally transforming cancer theranostics through the precise creation of multifunctional nanoparticles. Key nanomaterials like liposomes, gold and iron oxide particles, and quantum dots will take advantage by using machine learning (ML) and AI to guess how drugs work, find tumors better, and keep healthy cells safe. Along this line, AI is transforming healthcare by shifting medicine from a “one-size-fits-all” approach to highly personalized, predictive care. By analyzing complex multimodal data, AI enables clinicians to tailor treatments to an individual’s unique genetics. ML algorithms rapidly sequence and interpret genomic, transcriptomic and metabolomic data to uncover unique molecular disease drivers at any stage of disease progression. Advanced computing detects subtle digital biomarkers in medical imaging and wearable device data. This allows for the early detection and profiling of complex conditions long before clinical symptoms appear. Algorithms simulate how specific patient subsets will respond to treatments. This minimizes trial-and-error prescribing, optimizes drug dosages, and significantly reduces adverse side effects. While the convergence of AI and precision medicine promises to revolutionize healthcare, the field faces several real-world hurdles. Core challenges currently being addressed include integrating siloed data, protecting patient privacy, and preventing algorithmic bias [166]. Indeed, AI is transforming the study and clinical management of the NVU. AI accelerates this field through advanced diagnostic imaging, precision drug delivery and the predictive modeling of NDs. AI will help to highlight the integration of biomolecular tools and nanomaterial-based engineering approaches to facilitate the targeted delivery of theranostic agents to the brain [167]. Combining multiomics data with artificial intelligence transforms drug discovery by decoding complex biological systems, speeding up target identification, and improving clinical trials. While challenges like data integration and bias remain, this fusion revolutionizes translational medicine across oncology and neurology [168]. Nucleotide–peptide and peptide–drug conjugates are advanced hybrid therapies that combine payloads with peptide scaffolds to create dual-action treatments. This promising theranostic approach once again depends on improving bioconjugation chemistry, stability, and clinical translation using new chemical strategies, AI-driven design tools, and hybrid nanostructures [169]. Moreover, AI will improve BBB penetration and central nervous system drug delivery by predicting molecular permeability, designing targeted viral or nanocarrier delivery systems, and optimizing real-time physical interventions like focused ultrasound. These ML tools will drastically reduce the time needed to screen and engineer safe treatments for brain disorders. A ten year forecasting roadmap outlines three phases: AI nanoparticle design, digital twins, and clinical use. Key challenges will include toxicity, data rules, and the need for clinical testing.

The intersection of AI, ML and robotics together will offer transformative potential in designing intelligent innovative theranostic strategies capable of efficient and safe drug targeting in the diseased brain. Collectively, this perspective will serve as a consolidated starting point for changing clinicians’ minds, biomedical scientists’ and technologists’ views aiming to develop innovative strategies for precision medicine-oriented therapies and breakthrough biopharmaceutical applications.

## Figures and Tables

**Table 1:** Physiological role of NVU cell types.

Cell Type	Main Physiological Functions
Endothelial cells	BBB integrity
	Selective permeability
	Molecular transport
Pericytes	BBB stabilization
	CBF regulation
	Glymphatic clearance
Astrocytes	Neurovascular coupling
	Glutamate homeostasis
	TJ formation
	Metabolic support
Microglia	BBB surveillance
	Immune regulation
	Tissue repair
Neurons	Neurovascular communication CNS signaling

BBB = blood–brain barrier, CBF = cerebral blood flow, TJ = tight junction. CNS = central nervous system.

**Table 2:** Alterations affecting the different NVU components across neurodegenerative diseases and aging.

Cell Type	Alzheimer’s Disease	Parkinson’s Disease	Huntington’s Disease	Amyotrophic Lateral Sclerosis	Aging
Endothelial cells	Loss of TJs/AJs; ↑ MMP-2; ↑ MMP-9; ↓ LRP1; ↑ RAGE-mediated influx pathway.	↓ Mitochondrial function; ↑ ROS; NF-κB activation; ↓ TJ; ↑ AJs.	↑ mHTT endothelial deposition; ↑ Drp1- mitochondrial fission; ↑ ROS; ↓ NO; ↓ GLUT1.	↑ TDP-43/FUS endothelial accumulation; ↓ Expression of TJ proteins; Basement membrane remodeling.	p16/p21 activation; Release of inflammatory mediators; ↑ MMPs and coagulation factors; eNOS uncoupling; Mitochondrial dysfunction.
Pericytes	Pericyte loss; ↑ Cyclophilin A- inflammatory pathways; ↓ CBF and glymphatic system; ↓ Clearance of Aβ and τ species.	↑ Contractile dysfunction; ↓ Vascular coverage induced by α-syn accumulation.	↑ Cellular stress induced by disruption of mitochondrial dynamics.	Pericyte degeneration associated with vascular rupture.	Fibrogenic phenotype; ↓ PDGF-BB signaling; ↓ Capillary tone modulation in response to neuronal activity.
Astrocytes	↓ Glutamate uptake and potassium buffering; Disruption of gap junction; ↓ Support of neuronal metabolism; Loss of endfeet AQP4 polarization.	Involved in the exosome-mediated transfer of α-syn; ↓ Autophagy; ↓ Neurotrophic support; ↓ GLT-1 transporter; ↑ Release of proinflammatory mediators.	↑ mHTT expression; ↓ GLT-1 transporters and impaired glutamate clearance; ↓ Support of neurons’ energy demands.	TDP-43 and FUS astrocytic accumulation; ↑ Release of ROS, aberrant lipid metabolites, inflammatory cytokines and mutant protein species.	↓ Glutamate uptake and potassium buffering; ↓ Metabolic support; ↓ Maintenance of water homeostasis; ↓ AQP4 polarization.
Microglia	Promote maladaptive inflammatory responses; ↑ Production of TNF-α, IL-1β, IL-6, ROS and complement factors.	Facilitate the activation of the central and peripheral inflammation, amplifying neuronal injury.	Proinflammatory phenotype in the prodromal stage of the disease.	Chronically active; Contribute to BSCB altered permeability and disruption.	↓ Phagocytic activity; ↑ Lysosomal dysfunction; ↑ Inflammasome pathways; ↓ Threshold for pathological responses.
Neurons	NVU disruption is an active contributor to disease pathogenesis and represents one of the earliest drivers of neuronal degeneration.	Loss of dopaminergic neurons in the substantia nigra pars compacta; NVU alterations resulting from direct α-syn pathology contribute to the disease progression.	Chronic NVU metabolic deficit and neuroinflammation affect the vulnerable striatal medium spiny neurons, promoting their loss.	NVU dysfunctions increase motor neuron vulnerability by promoting excitotoxicity by a reduced secretion of trophic factors and by sustaining inflammation.	↑ Vasculature alterations; ↑ White matter hyperintensities; Enlargement of perivascular spaces; Barrier dysfunction; ↓ Clearance of toxic molecules.
Ref.	[20,23,24,25,26,27,28,29,30,31,32,33,35,36,37,39,40,41,42,43,44,46,47,48,49,50]	[57,58,59,60,61,62,63,64,65,66,67,68,69,70,71,72,73,75,76,77,78,79,80,81,82,83]	[101,102,103,104,105,106,121]	[43,85,86,87,88,89,90,91,92,93,94,95,96,97]	[107,108,109,110,111,112,113,114,115,116,117,118,119,120]

AJ = adherence junctions, MMP = matrix metalloproteinases, CBF = cerebral blood flow, BSCB = blood–spinal cord barrier, AQP4 = aquaporin-4, ROS = reactive oxygen species, NO = nitric oxide, eNOS = endothelial nitric oxide synthase; “↑” = upregulation/increase, “↓” = downregulation/decrease, Ref. = References.

## Data Availability

No new data were created or analyzed in this study. Data sharing is not applicable to this article.

## Abbreviations

The following abbreviations are used in this manuscript:

Aβ	Amyloid-β
AD	Alzheimer’s disease
AI	Artificial intelligence
ALS	Amyotrophic lateral sclerosis
AQP4	Aquaporin-4
BACE1	β-site APP-cleaving enzyme 1
BBB	Blood–brain barrier
Bcl-2	B-cell lymphoma 2
BSCB	Blood–spinal cord barrier
CBF	Cerebral blood flow
CNS	Central nervous system
DALY	Disability-adjusted life year
eNOS	Endothelial nitric oxide synthase
EVs	Extracellular vesicles
EXs	Exosomes
FUS	Fused in sarcoma
GLRX	Glutathione-dependent disulfide oxidation reduction reaction catalyzing protein
GWAS	Genome wide association study
HD	Huntington’s disease
HTT	Huntingtin
iPSC	Induced pluripotent stem cell
LNPs	Lipid nanoparticles
mHTT	Mutant huntingtin
miRNAs	microRNAs
ML	Machine learning
MMP	Matrix metalloproteinase
MNRs	Micro-/nanorobots
MRI	Magnetic resonance imaging
NDs	Neurodegenerative diseases
NPs	Nanoparticles
NVU	Neurovascular unit
PD	Parkinson’s disease
RMST	Rhabdomyosarcoma 2-associated transcript
TDP-43	TAR DNA-binding protein 43
